# Case Report and Review of the Literature: Bullous Skin Eruption After the Booster-Dose of Influenza Vaccine in a Pediatric Patient With Polymorphic Maculopapular Cutaneous Mastocytosis

**DOI:** 10.3389/fimmu.2021.688364

**Published:** 2021-07-15

**Authors:** Davide Sarcina, Mattia Giovannini, Teresa Oranges, Simona Barni, Fausto Andrea Pedaci, Giulia Liccioli, Clementina Canessa, Lucrezia Sarti, Lorenzo Lodi, Cesare Filippeschi, Chiara Azzari, Silvia Ricci, Francesca Mori

**Affiliations:** ^1^ Allergy Unit, Department of Pediatrics, Meyer Children’s University Hospital, Florence, Italy; ^2^ Dermatology Unit, Department of Pediatrics, Meyer Children’s University Hospital, Florence, Italy; ^3^ Immunology Unit, Department of Pediatrics, Meyer Children’s University Hospital, Florence, Italy

**Keywords:** vaccination, cutaneous mastocytosis, prevention, premedication, adverse reaction, pediatrics

## Abstract

Vaccination is a well-known trigger for mast cell degranulation in subjects affected by mastocytosis. Nevertheless, there is no exact standardized protocol to prevent a possible reaction after a vaccine injection, especially for patients who have already presented a previous vaccine-related adverse event, considering that these patients frequently tolerate future vaccine doses. For this reason, we aim to share our experience at Meyer Children’s University Hospital in Florence to raise awareness on the potential risk for future vaccinations and to discuss the valuable therapeutic strategies intended to prevent them, taking into account what is proposed by experts in literature. We describe the case of an 18-month-old female affected by a polymorphic variant of maculopapular cutaneous mastocytosis that presented an extensive bullous cutaneous reaction 24 hours after the second dose (booster dose) of inactivated-tetravalent influenza vaccine, treated with a single dose of oral corticosteroid therapy with betamethasone (0.1 mg/kg) and an oral antihistamine therapy with oxatomide (1 mg/kg/daily) for a week, until resolution. To the best of our knowledge, in the literature, no documented case of reaction to influenza vaccine in maculopapular cutaneous mastocytosis is described. Subsequently, the patient started a background therapy with ketotifen daily (0.05 mg/kg twice daily), a non-competitive H1-antihistamine, and a mast cell stabilizer (dual activity). A non-standardized pharmacological premedication protocol with an H1-receptor antagonist (oxatomide, 0.5 mg/kg) administered 12 hours before the immunizations, and a single dose of betamethasone (0.05 mg/kg) together with another dose of oxatomide (0.5 mg/kg) administered 2 hours before the injections was followed to make it possible for the patient to continue with the scheduled vaccinations. Indeed, no reactions were subsequently reported. Thus, in our experience, a background therapy with ketotifen associated with a premedication protocol made by two doses of oxatomide and a single dose of betamethasone was helpful to make possible the execution of the other vaccines. We suggest how in these children, it could be considered the idea of taking precaution when vaccination is planned, regardless of the kind of vaccine and if a dose of the same vaccine was previously received. However, international consensus needs to be reached to manage vaccinations in children with mastocytosis and previous adverse reactions to vaccines.

## Introduction

The term “mastocytosis” defines a various group of disorders characterized by an increase of mast cells in cutaneous tissue and different organs such as bone marrow, spleen, liver, lymph nodes, and gastrointestinal tract (1, 2). The organs most frequently involved are skin and bone marrow (2, 3). Mast cells’ uncontrolled proliferation is mainly associated with gaining function mutations in c-KIT, the gene that encodes for a tyrosine kinase receptor expressed by mast cells (4, 5). Disease manifestations are mostly linked to mast-cell-derived mediators’ release, especially histamine, and include pruritus, flushing, gastrointestinal signs and symptoms, and rarely anaphylaxis (6, 7). The typical Darier’s sign refers to the development of a wheal-and-flare reaction after a few minutes of scratching or rubbing a cutaneous lesion, due to the release of vasoactive mediators (8).

The current classification of mastocytosis, recently updated by World Health Organization in 2016, identifies three major disease groups: cutaneous mastocytosis, systemic mastocytosis, and mast-cell sarcoma (9, 10).

The classification of cutaneous mastocytosis is subcategorized based on the macroscopic features of the skin lesions, their distribution, and onset into three groups: maculopapular cutaneous mastocytosis (MPCM), formerly known as urticaria pigmentosa (furtherly sub-dived in monomorphic and polymorphic types), diffuse cutaneous mastocytosis (DCM), and mastocytoma of the skin (3, 9).

In children, mastocytosis is commonly limited to the skin, has a favorable prognosis, and improves spontaneously by the mid-teenage years. On the contrary, adult mastocytosis is usually systemic and may have a chronic or aggressive course (7, 9, 11).

The most frequent type of pediatric mastocytosis is MPCM, specifically the polymorphic variant, followed by the cutaneous isolated mastocytoma and then the diffuse cutaneous mastocytosis (9). Mastocytosis can appear at all ages but, it is possible to identify two incidence peaks: early childhood (65%) and young adulthood (35%). Focusing on the pediatric age, about 15% are connatal forms, 30% appear in the first 6 months of life, and the others between 2 and 15 years (7).

Patients affected by mastocytosis present a greater risk than the general population for anaphylaxis. The main cause of anaphylaxis in pediatric mastocytosis is idiopathic, followed by some stimuli such as foods, medications, and Hymenoptera venoms. The latter ones represent a rare cause of anaphylaxis in pediatric mastocytosis, while they represent the most common triggers in adult patients. In pediatric mastocytosis, the risk of anaphylaxis is related to the extent and the density of lesions. Elevated basal serum tryptase levels, systemic disease, KIT D816V mutation, and previous episodes of anaphylaxis represent other suggested risk factors. For this reason, it can be considered reasonable to provide these children with extensive disease and/or previous anaphylaxis with an emergency autoinjector of epinephrine (12, 13).

Vaccination is a well-known trigger for mast cell degranulation in subjects affected by mastocytosis. Nevertheless, there is no exact standardized protocol to prevent a possible reaction after a vaccine injection, especially for patients who have already presented a previous vaccine-related adverse event, considering that these patients frequently tolerate future vaccine doses. For this reason, we aim to share our experience at Meyer Children’s University Hospital in Florence to raise awareness on the potential risk for future vaccinations and to discuss the valuable therapeutic strategies intended to prevent them, taking into account what is proposed by experts in literature.

## Case Report

**Figure 1:**
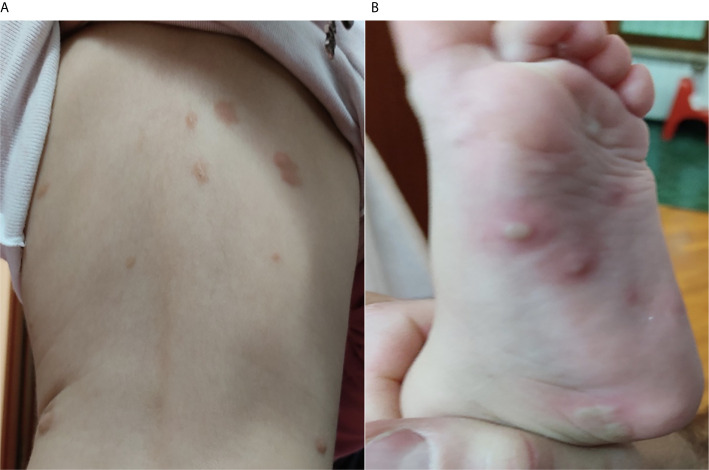
**(A)** Initial bullous skin eruption involving the back of the patient after 24 hours from the second dose of the influenza vaccine; **(B)** Bullous lesions presented on the left foot of the patient after 24 hours from the second dose of the influenza vaccine.

A female child of 18 months, affected by a polymorphic variant of MPCM, was presented to our attention following a significant diffuse bullous cutaneous reaction 24 hours after the injection of the second dose (booster dose) of the inactivated tetravalent influenza vaccine **(** **Figure 1** **)**.

Before this event, the patient did not present any cutaneous reactions to the injection of the other vaccinations scheduled in Italy for this age group, although fever was frequently reported after most of these immunizations (**Table 1**).

**Table 1:** Vaccines received by the patient in accordance to the Italian scheduled vaccinations.

Vaccines	Number of doses	Adverse clinical manifestations
Hexavalent vaccine	3	Fever^*^
Meningococcal B vaccine	3	Fever^*^
Rotavirus vaccine	3	Fever^*^
Meningococcal C vaccine	1	Fever^*^
Pneumococcal vaccine	2	Fever^*^

*Fever arisen 6–8 hours after each dose. Influenza vaccine is highly recommended in Italy. For children under 9 years of age who have not previously been vaccinated against influenza, a second dose should be administered after an interval of at least 4 weeks.

Hence, the patient was addressed to our center with the primary purpose of continuing the scheduled vaccinations in a hospital clinical setting. In her family history, no other cases of mastocytosis were reported. The older sister was affected by atopic dermatitis, and the mother had a history of reaction to penicillins. No other significant disorders, except for mastocytosis, were mentioned in her personal medical history.

At the age of 8 months, a first isolated mastocytoma appeared on the neck followed by more lesions, heterogeneous in shape and size, brownish-yellowish, on the head, chest, back, and legs. Sometimes, blisters occurred within the existing lesions. The diagnosis of polymorphic MPCM was made at the age of 9 months, after a dermatological evaluation that pointed out the characteristic skin lesions. During the physical exam, a gentle rubbing of a chest lesion revealed a prominent Darier’s sign associated with flushing of the face, neck, and chest. The serum tryptase level subsequently evaluated was within normal limits at 4.1 mcg/L. No skin biopsy was performed.

At the age of 13 months, the patient received her first dose of influenza vaccine, and only a 2-day fever with no cutaneous involvement was reported. One month later, the patient received the second dose of inactivated-tetravalent influenza vaccine, and after 24 hours from the injection, she presented an extensive bullous skin eruption involving the face, head, chest, shoulder, back, hands, and feet **(** **Figure 1** **)**. No blisters were present before the immunization. Moreover, she showed significant irritability and generalized pruritus. The adverse reaction was promptly treated with a single dose of oral betamethasone (0.1 mg/kg) and an oral antihistamine therapy with oxatomide (1 mg/kg/daily) for a week, with the complete resolution of the lesions.

After 6 days from the resolution of the clinical manifestations, another evaluation of serum tryptase level was made, showing results within normal limits (2 mcg/L). After this adverse reaction, parents refused the next Italian scheduled vaccination with the third dose of 13-valent Pneumococcal conjugate vaccine (PCV13). The patient was later addressed by our Allergy Unit thanks to the parents’ desire to catch up with the scheduled vaccination in a hospital clinical setting.

After a clinical evaluation of the patient, we planned the third dose of the PCV13 after 2 weeks from the first visit. Until that day, the patient assumed a background therapy with ketotifen (0.05 mg/kg twice daily), a non-competitive H1-antihistamine, and a mast cell stabilizer (dual activity). Furthermore, 12 hours before the injection, the patient assumed oxatomide (0.5 mg/kg), and 2 hours before the injection, she assumed the same dose of oxatomide together with a single dose of betamethasone (0.05 mg/kg). We decided not to perform a patch test to evaluate a possible delayed reaction to a component of the vaccine because of the possibility of provoking mastocytes degranulation. After 2 hours of observation from the injection, the patient did not present any cutaneous or systemic clinical manifestations. The patient was discharged with instructions to continue the therapy with ketotifen (0.05 mg/kg twice daily).

Afterward, the patient continued the same background therapy with ketotifen until the varicella vaccination, planned after 1 month. Similarly to the previous vaccination, the patient assumed oxatomide (0.5 mg/kg) 12 hours before the immunization and the same dose of oxatomide together with a single dose of betamethasone (0.05 mg/kg) 2 hours before the immunization. After the immunization, no adverse reactions were presented, including fever.

## Discussion

MPCM, the most common form of mastocytosis seen in childhood, is subdivided into two variants: polymorphic and monomorphic (9).

In the polymorphic variant, lesions are larger and asymmetric, and they have a typical distribution that affects the head, neck, and extremities. The lesions can be red, brown, or yellow and appear flat or elevated with sharp or indistinct margins. Darier’s sign, pruritus, and dermographism are usually present, while blistering may occur, especially in children under 3 years old. Serum tryptase levels are usually in the normal range; rarely patients with pronounced lesions can present increased level that usually decreases within 1 or 2 years (8, 14). The prognosis is excellent, and usually, lesions resolve by adolescence (2).

On the other side, the monomorphic variant is typical of adult age. The lesions are usually smaller, round, and similar. In these patients, serum tryptase levels can be persistently increased over time, and the disease tends to persist into adulthood with a higher risk of developing systemic mastocytosis (3, 8, 14).

Mastocytoma is the second most common lesion in the pediatric population. It usually occurs in the form of a single lesion, yellow-brownish, with the frequent formation of pomphoid lesions, the possible formation of bullous skin eruptions, and regional or diffuse flushing after the mechanical stimulation of the lesion. It has a dynamic pattern and can increase by dimensions (but not number) and usually regress by mid-teenage years (14, 15). According to Hartmann et al., a maximum of three compatible lesions still should qualify as mastocytoma, while more than three lesions should be considered as attributable to a case of MPCM (3). The diagnosis is based exclusively on clinical manifestations, and serum tryptase levels are usually within the normal range (3, 14).

DCM is characterized by diffuse infiltration of the dermis with mast cells, which results in generalized erythema and diffuse papules associated with pachydermia, darker skin, and accentuation of hair follicles “peau d’orange” (16). Dermographism and blistering are characterizing signs of disease (3, 8). Diagnosis is made by skin biopsy (16). DCM can resolve by adolescence, but sometimes progress toward systemic mastocytosis can be seen, usually accompanied by hepatomegaly, splenomegaly, and bone marrow involvement (3, 8).

With a complete inspection of the skin accompanied by Darier’s sign evaluation, the physical examination is critical to have an initial approach to the disease. A skin biopsy that shows evidence of compatible histological findings can confirm the diagnosis, even if, in the absence of them, a diagnosis cannot be ruled out (17). As concerns the laboratory test, a significant role is played by the serum level of tryptase. In cutaneous mastocytosis and isolated mastocytoma, serum levels of tryptase are usually within the standard limit. If a tryptase serum level >20 mcg/L is detected, without signs and symptoms of systemic involvement, the value should be considered in first place associated with a release of mediators by cutaneous mast cells. The evaluation of serum levels is indeed recommended after 24 hours from the resolution of a clinical event caused by mast cells degranulation. Anyway, periodic control of tryptase should be considered in order to rule out a progression of disease (18). The study of c-KIT mutation should be carried out in selected patients (8), while a bone marrow biopsy should be requested if systemic clinical manifestations, especially organomegaly, in conjunction with elevation of tryptase level are present (9, 19).

Multiple pathways have been described in the activation and degranulation of mast cells. One important signaling pathway is induced by the high-affinity receptor for IgE, and it is involved in immediate allergic reactions. Other triggers for mast cell activation are known, and they involve certain cytokines, immune complexes, complement proteins, drugs (e.g., non-steroidal anti-inflammatory drugs and opioids), radiocontrast media, products of bacteria, or parasites. Physical factors such as heat, cold, friction, stress, and physical effort can induce mast cell activation and act as cofactors in allergic and anaphylactic reactions (20, 21). In our experience, we indeed avoided these known cofactors of mast cell degranulation. The vaccine was kept out of the fridge for 2 hours to bring it to room temperature, and the injection was carefully carried out as not to rub the skin. The patient was quiet, and we knew that she was not assuming other medications, except for the ones that we prescribed, and that she did not have histamine liberators food.

Vaccines may act as triggers in patients with mastocytosis, especially children. Indeed, some substances contained in vaccines, such as dextran, gelatin, and polymyxin B, may be responsible for this adverse reaction (22). However, literature does not specifically describe the pathway associated with mastocyte degranulation after immunization.

An Italian study of 102 patients, including 35 children and 67 adults with mastocytosis, demonstrated no relationship between types of vaccines and risk of mast cell degranulation after immunization. Seven children in the study presented reactions after the immunization. Subsequently, some of these children tolerated the same vaccine components, strengthening the hypothesis on the possible role of variable triggers in determining reactions to vaccines in cases of mastocytosis (**Table 2**) (22).

**Table 2:** Clinical data about patients from the analyzed studies on vaccination and mastocytosis.

Authors	Patients with reactions/Total sample	Variant of Mastocytosis/Age of Diagnosis (years)	Eliciting Vaccine (number of doses received)	Reaction/Time interval	Therapy	Subsequent Vaccines/Premedication
Zanoni et al. (22)	7/102 (35 children and 67 adults)	Mastocytoma/0.5	Hexavalent (1)	Urticaria and angioedema/20 min	Unknown symptomatic treatment	DTaP, IPV, HB, Hib, MMRV/not available
		Mastocytoma/0.4	Hexavalent (2)	Local and facial flushing/20 min		Hexavalent/not available
		Mastocytoma/2	MenC (1)	Fever and gastrointestinal clinical manifestations/24 h		PCV/not available
		Mastocytoma/0.6	Hexavalent (3)	Injection site reaction and fever/8 h		
		MPCM/0.2	PCV (2), MenC (1)	Fever/8 h		
		MPCM/0.5	HPV (1)	Hives on arm and nasal obstruction/12 h		
		DCM/0.4	Hexavalent (1, 2) PCV (1, 2)	Hives and itch on trunk and febrile convulsions/12 h		
Parente et al. (6)	4/72 children	MPCM/0.3	Hexavalent (1)	Bullous skin reaction/6–12 h	Oral antihistamine	Other mandatory vaccines/oral antihistamines
		Mastocytoma/2	Hexavalent (1)	Diffuse urticaria/1–4 h	Oral antihistamine	Other mandatory vaccines
		Mastocytoma/4	Hexavalent (1)	Diffuse urticaria/1–4 h	Oral antihistamine	Other mandatory vaccines
		DCM/0.1	Hexavalent (1)	Bullous skin reaction and mild bronchospasm/6–12 h	Nebulized epinephrine and oral antihistamine	Other mandatory vaccines/oral antihistamines
Bankova et al. (16)	1 child	DCM	DTaP IPV HiB PCV Rotavirus	Confluent blisters on the back, abdomen, and upper arms/a day later	5-day course of low-dose oral steroids	Unknown/oral and topical sodium cromolyn
Johansen et al. (23)	3/35 children	MPCM	DTaP IPV HiB PCV Rotavirus	Skin flushing, itch, blisters, gastrointestinal clinical manifestations/hours	Oral antihistamine	
		MPCM	DTaP	Skin flushing and pruritus/minutes	Oral antihistamine	
		MPCM	All vaccines	Fever/hours	Acetaminophen	

Hexavalent, diphtheria–tetanus toxoid–acellular pertussis (DTaP)–hepatitis B (HB)–inactivated polio vaccine (IPV)–Haemophilus influenzae B vaccine (HiB); h, hours; HPV, human papilloma virus vaccine; IPV, inactivated polio vaccine; min, minutes; Men C, meningococcal C vaccine; MMRV, measles, mumps, rubella, varicella vaccine; PCV, pneumococcal vaccine.

Parente et al., supposed that mast cell activation could be induced by a component in vaccines that acts like a superantigen, which unspecifically binds to human IgE, leading to mast cell degranulation. Furthermore, they speculated that the development of IgG or IgM after the first dose of vaccine, which neutralizes the superantigens before they interact with IgE, could explain the lack of reaction to the booster vaccination.

This possible role of variable triggers in determining reactions to vaccines in cases of mastocytosis is not deeply analyzed in the literature. Additionally, when a specific trigger was identified, its role in the underlying mechanisms of reactions and cofactors could not be explained. For that reason, in the future, it will be of great interest to analyze the role of possible cofactors in cases of reactions to vaccines in patients with mastocytosis.

Parente et al., in their study of the evaluation of vaccination safety in 72 pediatric patients with mastocytosis, concluded that vaccine reactions in mastocytosis are usually mild and do not generally occur with boosters (as confirmed by Hussain et al.) (**Table 2**) (6, 9). After the first dose of the hexavalent vaccine, four patients presented cutaneous reactions. All four of the children who presented a vaccine-related reaction received the subsequent vaccinations and boosters in a monitored hospital setting. Among them, the two patients who presented a previous bullous reaction assumed oral antihistamines beginning 48 hours before vaccine administration. Moreover, the patient affected by DCM was on a continuous antihistamine treatment. None of the four children presented a further reaction to the subsequent two boosters of hexavalent or the other mandatory vaccines, which may demonstrate a potential role for antihistaminic treatment in preventing further reaction. Hence, Parente et al., propose to limit a prolonged observing time to 2 hours, only in case of administration of the first dose of vaccine (6). In our experience, considering the significant reaction that occurred on the booster dose of a previous vaccine and in contrast to scientific literature that defines how adverse events usually occur with the first dose of vaccine, we found it difficult to stigmatize reaction to vaccination in mastocytosis, and so we decided to extend the 2-hours observation time for all the vaccines administered (6). Parents should be trained to identify the warning signs of an early adverse reaction and taught how to use an epinephrine autoinjector when necessary (12, 22). In the analyzed study by Parente et al., hexavalent vaccines (and therefore, polyvalent formulation) seem to be associated with a higher risk of reaction (6). Nevertheless, there is no significant overwhelming scientific evidence in the literature to suggest avoiding polyvalent formulation. Taking into account ministerial Italian recommendations (24) and other sources of information (16, 22), would suggest avoiding the co-administration of multiple vaccines (22) and not the use of polyvalent vaccines, also for practical reasons, including the maximization of the adherence to scheduled vaccinations. We decided to administer varicella vaccine in a single injection, instead of measles, mumps, rubella, and varicella vaccine polyvalent formulation, only for the wish of patient’s family, that was still frightened and distrustful of vaccines. It is indeed very important that families regain their trust in vaccinations, especially among these kinds of patients and in the anti-vaccination movement era (25).

A recent retrospective study of 35 patients evaluated vaccination reactions in pediatric patients with MPCM (**Table 2**) (23). The study’s conclusions are similar to previous ones and highlight that vaccination reactions in mastocytosis tend to be mild, so scheduled vaccinations should be continued (23).

Bankova et al., highlight the importance of precautions with procedures and adequate premedication to reduce the risk of severe complications. The authors describe a case of generalized bullous cutaneous reaction in a 5-month-old boy affected by DCM after the scheduled 4-month vaccinations (**Table 2**). In this case, the patient was treated with a low-dose oral steroid for 5 days, which was indicated by the authors as the preferred therapy for cases with blister formation according to indications reported in the literature. Subsequently, the patient presented two additional episodes of blistering associated with vaccination and viral illness but, these episodes became milder, thanks to the initiation of a regimen of oral and topical sodium cromolyn (16).

## Conclusion

The lack of specific guidelines to follow in case of vaccination reaction in cutaneous mastocytosis represents a significant issue to face. The lack of predictive elements of the risk of reaction makes it impossible to define its level for future vaccinations, even if the literature states that these patients frequently tolerate future vaccine doses. Furthermore, there is a lack of consensus on which therapy is most appropriate. In addition, there are no criteria to define a stratification of risk that allows us to identify who is the most deserving patient to receive it. It would be very important to collect extensive multicentric data with the aim to compare different schemes of premedication to establish the most efficient protocol in the most suitable patient, but this is made difficult by the rarity of pathology.

In this case report, we are far from establishing the best therapeutic strategy to adopt, but we took into account and shared information collected from the literature to define a non-standardized pharmacological and procedural protocol, which we adopted in our experience. Thus, a background therapy with ketotifen associated with a premedication protocol made by two doses of oxatomide and a single dose of betamethasone was helpful to make possible the execution of the other vaccines. Though, as previously underlined, since the incidence of future reactions appears unpredictable, the child might have tolerated the subsequent vaccination without premedication, but we did not consider this a deterrent to not perform a premedication therapy considering the significant role of premedication described in literature in these patients to prevent and control adverse events and the patient’s family fear to execute the other mandatory vaccinations, especially so shortly after a relevant reaction. It is indeed important to highlight as well the significant role played by the premedication therapy to make the patient’s family trust vaccinations again, considering that the patient subsequently did not present in the two vaccinations executed in our center any kind of clinical manifestations.

The choice to use betamethasone in our premedication protocol can be justified by the long-time latency between vaccine injection and clinical manifestations, so to control any delayed reactions. We avoided all the possible stimuli of reaction, and we adopted specific measures of care, as explained in literature (16, 21, 22). Finally, we suggest how in these children, it could be considered the idea of taking precaution when vaccination is planned, regardless of the kind of vaccine and if a dose of the same vaccine was previously received. However, international consensus needs to be reached to manage vaccinations in children with mastocytosis and previous adverse reactions to vaccines.

## Data Availability Statement

The original contributions presented in the study are included in the article/supplementary material. Further inquiries can be directed to the corresponding author.

## Ethics Statement

Written informed consent was obtained from the minor(s)’ legal guardian/next of kin for the publication of any potentially identifiable images or data included in this article.

## Author Contributions

CF and FM conceptualized the work. DS, MG, TO, FAP, SR, and FM drafted the manuscript. DS, MG, TO, SB, FAP, GL, CC, LS, LL, CF, CA, SR, and FM performed the investigations and critically revised the manuscript. All authors contributed to the article and approved the submitted version.

## Conflict of Interest

The authors declare that the research was conducted in the absence of any commercial or financial relationships that could be construed as a potential conflict of interest.
